# Can miRNA Indicate Risk of Illness after Continuous Exposure to *M. tuberculosis*?

**DOI:** 10.3390/ijms22073674

**Published:** 2021-04-01

**Authors:** Cleonardo Augusto Silva, Arthur Ribeiro-dos-Santos, Wanderson Gonçalves Gonçalves, Pablo Pinto, Rafael Pompeu Pantoja, Tatiana Vinasco-Sandoval, André Maurício Ribeiro-dos-Santos, Mara Helena Hutz, Amanda Ferreira Vidal, Gilderlanio Santana Araújo, Ândrea Ribeiro-dos-Santos, Sidney Santos

**Affiliations:** 1 PPG em Genética e Biologia Molecular, Laboratório de Genética Humana e Médica, Universidade Federal do Pará, Belém 66075-110, Brazil; cleonardoaugusto@gmail.cm (C.A.S.); arthurrdsantos@outlook.com (A.R.-d.-S.); wandersongegoncalves@gmail.com (W.G.G.); pablopintonet@yahoo.com.br (P.P.); rafaelpompeu988@gmail.com (R.P.P.); andremrsantos@gmail.com (A.M.R.-d.-S.); amandaferreiravidal@gmail.com (A.F.V.); gilderlanio@gmail.com (G.S.A.); sidneysantosufpa@gmail.com (S.S.); 2 PPG em Oncologia e Ciências Médicas, Núcleo de Pesquisas em Oncologia, Universidade Federal do Pará, Belém 66073-005, Brazil; 3 Laboratoire de Génomique et Radiobiologie de la Kératinopoïèse, Institut de Biologie François Jacob, CEA/DRF/IRCM, 91000 Evry, France; gtvinascos@gmail.com; 4 Instituto de Biociências, Departamento de Genética, Universidade Federal do Rio Grande do Sul, Porto Alegre 91501-970, Brazil; mara.hutz@ufrgs.br

**Keywords:** miRNA, tuberculosis, differential expression analysis

## Abstract

**Simple Summary:**

Tuberculosis is the leading cause of mortality from a single infectious agent and is among the top 10 causes of death worldwide. Despite that, few studies focus on regulatory elements such as small non-coding RNAs in tuberculosis. This pilot work applied Next Generation Sequencing techniques to evaluate the global miRNA expression profile of patients with active tuberculosis; their respective healthy physicians, who are at constant risk of infection; and a second group of healthy controls. In addition, we observed miRNA–gene interactions affected by exposure to the bacteria. Our findings indicate a list of miRNAs that could be used as potential biomarkers to improve treatment strategies at early stages. We also observed modified pathways related to the immune response due to differential miRNA expression profiles. Finally, we alert and encourage the development of new strategies to avoid long-term exposure of healthy physicians, considering how closely related their miRNA profile was to tuberculosis patients using current safety protocols.

**Abstract:**

The role of regulatory elements such as small ncRNAs and their mechanisms are poorly understood in infectious diseases. Tuberculosis is one of the oldest infectious diseases of humans and it is still a challenge to prevent and treat. Control of the infection, as well as its diagnosis, are still complex and current treatments used are linked to several side effects. This study aimed to identify possible biomarkers for tuberculosis by applying NGS techniques to obtain global miRNA expression profiles from 22 blood samples of infected patients with tuberculosis (n = 9), their respective healthy physicians (n = 6) and external healthy individuals as controls (n = 7). Samples were run through a pipeline consisting of differential expression, target genes, gene set enrichment and miRNA–gene network analyses. We observed 153 altered miRNAs, among which only three DEmiRNAs (*hsa-let-7g-5p*, *hsa-miR-486-3p* and *hsa-miR-4732-5p*) were found between the investigated patients and their respective physicians. These DEmiRNAs are suggested to play an important role in granuloma regulation and their immune physiopathology. Our results indicate that miRNAs may be involved in immune modulation by regulating gene expression in cells of the immune system. Our findings encourage the application of miRNAs as potential biomarkers for tuberculosis.

## 1. Introduction

Tuberculosis, a disease caused by *Mycobacterium tuberculosis* infection, is the leading cause of mortality from a single infectious agent and is among the top 10 causes of death worldwide, accounting for about 1.3 million deaths in 2017 and 10.4 million new cases of active tuberculosis in the world for that same year [1,2]. A third of the world population carries latent tuberculosis infections and, consequently, the potential of developing active tuberculosis [3]. HIV co-infection and age at first exposure are factors that contribute to the progression of the active form of the disease but explain only a fraction of the rate of activation of latent tuberculosis [4]. Disease progression may occur if host cell-mediated immunity fails, as suggested by the fact that an increased risk of latent tuberculosis reactivation is posed by anti-tumor necrosis factor therapy [5], which causes immune suppression, and the considerably high active tuberculosis prevalence among HIV-infected patients with T-cell immunity deficiency [5]. Identifying active tuberculosis high-risk groups would allow strategies for a more effective prophylactic treatment and would prevent the evolution of the disease to highly infectious symptomatic stages, avoiding transmission [6].

Gene regulation is an essential mechanism for both maintenance and normal functionality of the cell. Changes in gene expression may impact cellular state and even lead to complex and infectious diseases [7]. Non-coding RNAs (ncRNAs) are some of the main molecular elements that act in gene regulation. Among ncRNAs, miRNAs (small fragments of 18–22 nucleotides) are able to regulate the expression of genes at a post-transcriptional level by binding to the 3′-UTR of messenger RNAs (mRNAs) and inhibiting their translation or inducing their degradation [7,8].

miRNAs may regulate host response to tuberculosis, being potentially critical for the establishment of the infection. However, the dynamics of the miRNA expression and its implications for immune response in the lungs remains unknown [9]. Thus, considering their impact in cells, miRNAs have the potential to be used as biomarkers for diagnosis, response to treatment and therapeutic interventions [7].

Some studies also provide insight into the role of differentially expressed miRNAs (DEmiRNAs) in tuberculosis, such as discrimination of active tuberculosis and healthy controls [6,10,11] and the regulation of pathogen–host interactions [12,13,14]. However, there are few studies on the expression profile of miRNAs in tuberculosis using Next Generation Sequencing (NGS) techniques, which offer higher accuracy and sensitivity regarding miRNA sequences, allowing the identification of new and known miRNAs [15].

Risk prediction based on molecular biomarkers of individuals who will develop active tuberculosis within a population exposed to *Mycobacterium tuberculosis*, is extremely important [13]. Therefore, the aim of the present study was to apply NGS technology to evaluate the global miRNA expression profile (miRnome) of patients with active tuberculosis and to provide a brief overview of altered pathways regulated by these miRNAs using functional enrichment and miRNA–gene network analysis. Such strategies were implemented to identify regulatory elements which may, with further research, be used as biomarkers for early diagnosis of active tuberculosis.

## 2. Results and Discussion

In tuberculosis pathogenesis, host cellular immune response determines whether an infection becomes latent tuberculosis infection or progresses to active infectious or extrapulmonary tuberculosis [16]. It has been established that both adaptive and innate immune response is significantly regulated by miRNAs. miRNAs control the differentiation of B cells, antibody generation, T cell development [17] and function and innate immune cell activation [18]. Under mycobacterial infection, components of inflammatory and immune pathways are regulated by miRNAs [19,20].

The role of miRNAs in tuberculosis has been reviewed recently [21]. Pathways such as host inflammatory response and immune response signaling pathways, as well as a list of regulatory processes such as apoptosis, cytokine production, nitric oxide suppression, T cell proliferation, inhibition of antimicrobial peptides and autophagy, have been associated with miRNA regulation. It has been observed that lipid metabolism, a regulatory function associated with tuberculosis, can also be regulated by miRNAs [22].

### 2.1. RNA-Seq Data Overview

To identify potential biomarkers, miRNA sequence expression of 22 whole blood samples were analyzed. Samples were divided into three groups: External Control (n = 7); Hospital Control (n = 6), which consist of physicians responsible for tuberculosis treatment; and Tuberculosis patients (n = 9). Unfortunately, one sample belonging to the Tuberculosis patients group did not pass the quality control pipeline and was removed from the analysis.

RNA-seq data show an average of 3224 mapped reads per sample—ranging from 0 to 925,920 raw read counts. We identified, among 2576 known miRNAs, a total of 210 expressed miRNAs (i.e., miRNAs with total read count ≥ 10 in at least one sample). Of these, *hsa-miR-486-5p*, *hsa-miR-92a-3p* and *hsa-miR-16-5p* were the most abundant in all samples, representing about 82% of the total read count. These miRNAs were later removed from the analysis to avoid statistical bias.

**Figure 1:**
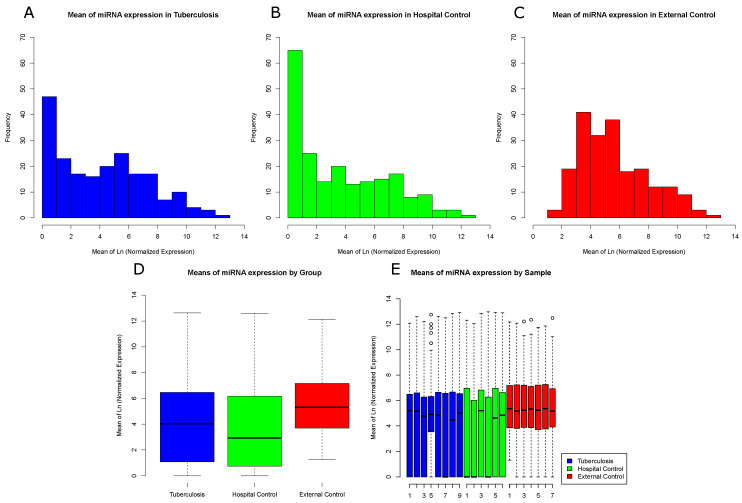
Frequency distribution of miRNA reads among sample groups: (**A**) log-normalized miRNA mean frequency distribution in Tuberculosis samples; (**B**) log-normalized miRNA mean frequency distribution in Hospital Control samples; (**C**) log-normalized miRNA mean frequency distribution in External Control samples; (**D**) log-normalized miRNA mean expression by groups; and (**E**) log-normalized miRNA mean expression by sample. One sample from the Tuberculosis patients group was filtered and removed due to low sequence quality.

Log-normalized mean frequencies for each miRNA and sample were used to generate histograms and boxplots of miRNA expression profiles. We found a clear distinction between the miRNA expression distribution in the External Control group and both Hospital Control and Tuberculosis groups, which showed a higher number of miRNAs with low expression levels (Figure 1A,B). Contrary to our initial belief, Hospital Controls had more miRNAs with low expression levels than Tuberculosis patients. The External Control group, as indicated by Figure 1C, had a higher expression profile compared to the other groups and presented few miRNAs with low expression values. High peaks of miRNAs expression are represented for each group and sample in Figure 1D,E, respectively.

We submitted miRNA expression data to statistical analyses to identify differential expression levels between group pairs and further investigate potential biomarkers and genetic mechanisms involved in the process of infection.

### 2.2. Differential Expression Analysis

Differential expression analysis process involves comparing pairs of groups (a case and a control group) to find statistically relevant differences between their miRNA expression profiles. Besides the three possible combinations of our three initial groups, two new comparisons were introduced to the study: (1) a merge of Hospital Controls and External Controls into a new Control group versus Tuberculosis patients (case group); and (2) a merge of Tuberculosis patients and Hospital Controls into a Medical Samples group (case group) versus the External Control group.

Among these five comparisons, two presented the most meaningful results and are further discussed: (i) Medical Samples versus External Controls; and (ii) Tuberculosis patients versus Hospital Control. Additionally, these results were used to perform both target genes analyses and gene set enrichment analyses.

#### 2.2.1. Medical Samples vs. External Control

This comparison poses an interesting discussion given it includes all sample groups and showed meaningful DEmiRNA results by providing an overview of similarities and distances between samples and sample groups.

**Figure 2:**
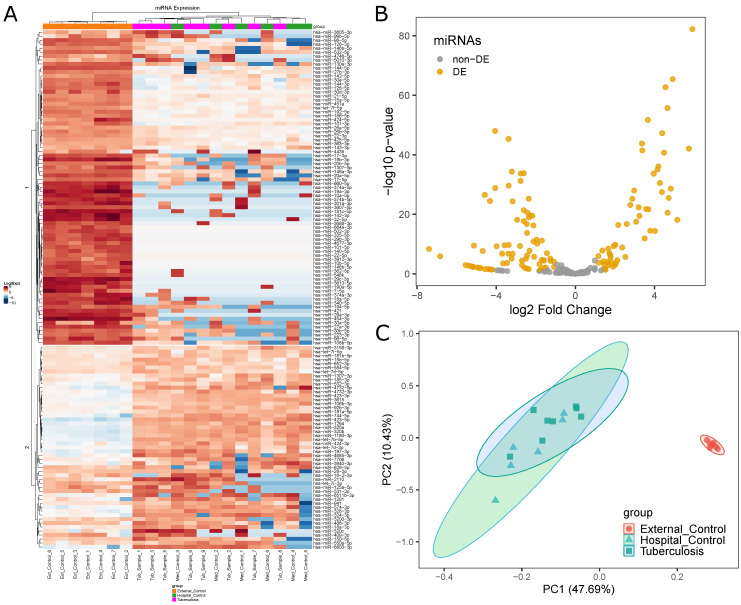
Differential miRNA expression analysis between Medical Samples and External Controls: (**A**) differences in miRNA expression levels between the three initial groups (EC, orange; HC, pink; TP, green) represented by a heatmap; (**B**) volcano plot highlighting 130 DEmiRNAs in Medical Samples versus External Controls; and (**C**) PCA plot showing distances between the three initial groups based on miRNA expression.

DEmiRNAs data obtained from edgeR showed differences in miRNA expression levels between the three initial groups (Figure 2A). This analysis is based on Kendall’s hierarchical clustering correlation of miRNA expression profiles in all samples and identified a clear distinction between External Control and Medical Samples groups. This contrast is presented in two clusters of DEmiRNAs. In the first cluster, the External Control group presented higher expression levels, while, in the second, External Controls presented lower expression levels. However, this comparison could not distinguish Tuberculosis patients from Hospital Controls.

In total, 130 miRNAs were found to be differentially expressed between Medical Samples and External Controls (Figure 2B). This was the second highest number of DEmiRNAs found in all five comparisons. While Tuberculosis versus External Controls yielded a higher number of DEmiRNAs (135), 122 of those were shared between both comparisons.

Principal Component Analysis (PCA) was performed to visualize how closely related the three initial groups were (Figure 2C) regarding their miRNA expression profile. The results show External Controls as a clearly separated cluster, while Tuberculosis patients largely overlapped with Hospital Controls. This visual representation provides insight into the similarities between the Hospital Control and Tuberculosis patients groups in a miRNA expression level.

#### 2.2.2. Tuberculosis Patients vs. Hospital Controls

This comparison pair provided the most notable expression results, given that Hospital Control group members have had continuous contact with Tuberculosis patients and, thus, have had constant exposure to *Mycobacterium tuberculosis*. We suggest that this durable exposure promotes changes in the organism, including miRNA expression profile. However, it seems there is a biological mechanism keeping members of the Hospital Control group from acquiring active tuberculosis. Results from differential expression analysis could potentially identify which miRNAs are regulating these mechanisms and use them as biomarkers for the disease.

**Figure 3:**
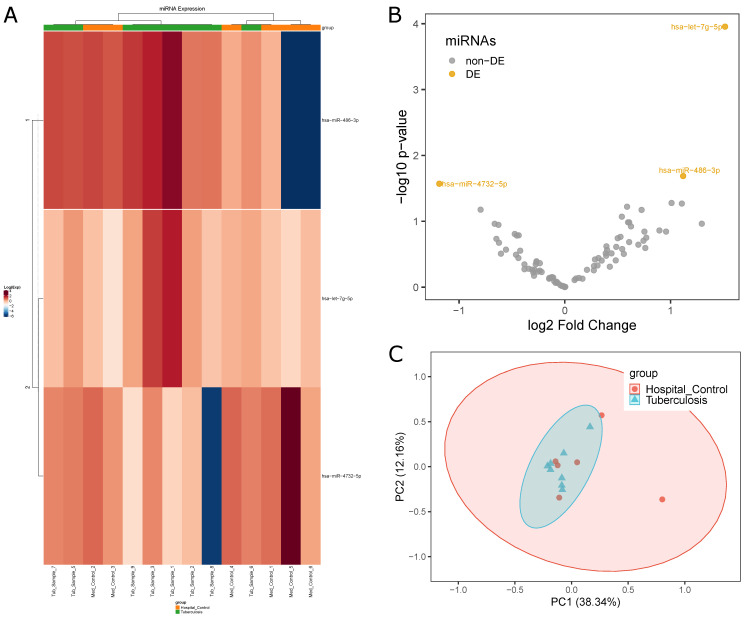
Differential miRNA expression profiles between Tuberculosis patients and Hospital Control samples: (**A**) difference in expression levels of miRNAs between the two groups (HC, orange; TP, green) indicated by a heatmap; (**B**) volcano plot highlighting three miRNAs with differential expression; and (**C**) PCA plot highlighting the homogeneity and separation of the groups based on miRNA expression.

The edgeR statistical analyses found only three DEmiRNAs (*hsa-miR-4732-5p*, *hsa-miR-486-3p* and *hsa-let-7g-5p*) (see Figure 3B), the first with higher expression in Hospital Controls and the last two with higher expression in Tuberculosis patients. This was the lowest number of miRNAs found in any of the performed comparisons, indicating a lack of DEmiRNAs involved in the process of keeping the organism safe. Both heatmap (Figure 3A) and PCA (Figure 3C) analyses did not reveal a clear distinction between the two groups. Results from this comparison are alarming considering both the small number of DEmiRNAs and the distances between the two groups being unexpectedly low, which indicated that Hospital Controls had been exposed to a higher risk level than previously expected.

### 2.3. Gene Set Enrichment Analysis

To perform this step, DEmiRNAs found in the previous analyses were submitted to both target gene analysis followed by functional enrichment analysis using Reactome online platform. Reactome requires a list of genes as input and generates a table including data from numerous pathways. Data from Medical Samples versus External Controls were run through a new quality control pipeline to boost target genes analysis. Enrichment results from both comparisons indicated that the DEmiRNAs may interact with cell proliferation inhibition and/or inflammatory response processes, which are some of the affected biological processes expected to have a role in tuberculosis disease. Enriched pathways were selected and classified according to regulatory functions involved in tuberculosis pathogenicity that could potentially be regulated by miRNAs.

Inflammatory response and apoptosis inhibition were reported by Chai et al. (2019) through targeting of FOXO1 [23]. Pro-inflammatory properties of Interleukin-6 were observed by Scheller et al. (2011) [24]. Bi et al. (2019) showed the suppression of cell proliferation associated to the inhibition of Egr1/TGF-β/Smad pathway by miRNA-181a-5p [25]. Spizzo et al. (2010) indicated a TP53 dependent proapoptotic regulatory loop [26]. Finally, Sabir et al. (2018) showed regulation of TLR and TNF by miRNAs [21]. Therefore, in accordance with the aforementioned research, we also investigated and identified enrichment of pathways related to FOXO, IL-6, TGF-β, TP53, TLR and TNF.

#### 2.3.1. Target Genes Analysis in Medical Samples vs. External Controls

Only DEmiRNAs with |log2FoldChange| (|FC|) > 2 were kept for this analysis, resulting in a total of 97 DEmiRNAs. Considering that miRNAs with positive FC values could potentially regulate different mechanisms of the human body compared to the miRNAs with negative FC values, these 97 DEmiRNAs were separated into two new groups, downregulated and upregulated. The downregulated group consisted of 59 DEmiRNAs, while the upregulated group included 38.

Target genes investigation revealed that 54 of 59 DEmiRNAs from the downregulated group presented validated experimental interactions with 948 different genes. The upregulated group presented 24 validated interactions with 221 genes, resulting in a total of 1051 genes. To identify which pathways were enriched by each group of DEmiRNAs, we performed a gene set enrichment analysis using Reactome.

Gene enrichment analysis for genes associated with upregulated miRNAs resulted in 1161 associated pathways, among which 346 had a significant association (FDR *p*-value ≤ 0.05). We found 1732 Reactome pathways regarding downregulated miRNAs (465 significant). Pathways related to tuberculosis were selected and their results were presented in Table 1 and Table 2, which contain pathway identification, number of interaction genes and FDR value. Each table was built to include pathways associated to either upregulated or downregulated miRNA group.

Intracellular microorganisms such as *Mycobacterium tuberculosis*, in interaction with the host, establish at least four stages of distinct cellular mechanisms: (i) recognition and engulfment; (ii) inflammation; (iii) apoptosis; and (iv) death of the pathogen [21]. These mechanisms involve several genes from various pathways, which act collectively at the time of infection to prevent the onset of the disease and try to defeat the pathogen. These pathways are under epigenetic regulation and the result of these parasite-host interactions can define the establishment of the disease. Table 1 and Table 2 show the main enriched pathways regulated by upregulated and downregulated miRNAs. Thus, the miRNA expression profile of the host in the face of infection or exposure to *Mycobacterium tuberculosis* indicates a mechanism capable of combating the bacillus. We can presuppose that these pathways may be relevant in helping to predict the point at which an endangered individual has their natural immune barriers circumvented by the pathogen.

#### 2.3.2. Target Genes Analysis in Tuberculosis Patients vs. Hospital Controls

Considering the low number of DEmiRNAs for this comparison (see Figure 3), the three DEmiRNAs (*hsa-miR-4732-5p*, *hsa-miR-486-3p* and *hsa-let-7g-5p*) were not divided into upregulated and downregulated groups and target genes analysis was performed. These DEmiRNAs had interactions with 78, 202 and 341 genes, respectively, and interacted with a total of 599 different genes. Interestingly, these DEmiRNAs showed interaction overlaps, as three genes, *CDKN1A*, *ZC3HAV1L* and *TUBB2A*, are common targets to all three DEmiRNAs.

A previous study revealed that *hsa-let-7g* contributed to apoptosis and loss of proliferation in gastric cancer cells under oxidative stress [27]. Additionally, this miRNA is reported to induce the increase of proliferation and reduction of apoptosis in normal cells by targeting antiangiogenic genes or apoptotic genes [28]. It is also suggested that the *let-7* miRNA family is involved in the regulation of anti-tuberculosis immune response [5].

Regarding the overlap among all three genes, studies show that *CDKN1A* may be involved in p53/TP53 mediated inhibition of cellular proliferation (G1 phase) in response to DNA damage [29,30]. *CDKN1A* encodes p21 protein, a member of Cip/Kip family. High levels of p21 have been previously reported in pulmonary sarcoidosis, and it was hypothesized that in these microenvironments the expression of p21 functions as an inhibitor for apoptosis and as a facilitator for the formation and maturation of granulomas [31,32,33]. *TUBB2A* encodes β-tubulin, a protein that plays an important role in cell division, migration and intracellular transport [34]. This gene could act in active macrophage, preventing mature phagolysosome formation and provide comfortable conditions for pathogen survival [35].

Both genes are related to inhibition of cellular proliferation and their regulation may be involved in the formation and maturation of granulomas and inhibition of phagolysosome, important steps for establishment of the disease.

### 2.4. Network Analysis of Target Genes

For this analysis, three different complex networks were built, namely two for interactions between miRNA–genes in Medical Samples versus External Controls and one for interactions in Tuberculosis patients versus Hospital Controls. Such networks were built regarding experimentally validated interactions catalogued in miRTArBase.

**Figure 4:**
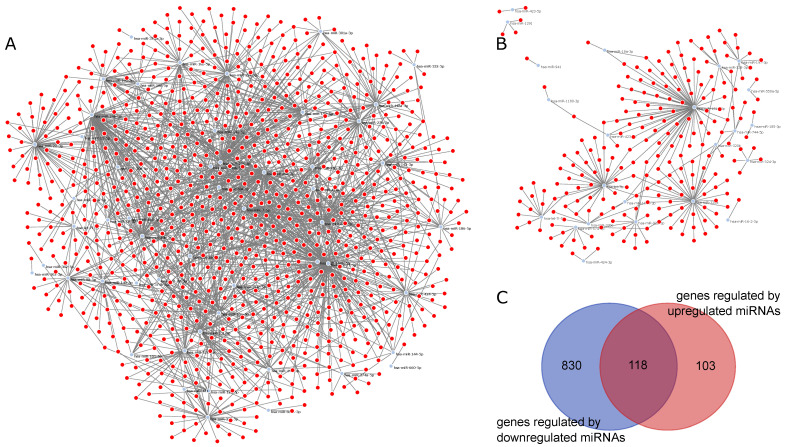
miRNA–gene networks for MS vs. EC generated by ncRNA-network tool: (**A**) graphical representation of the miRNA–gene network of downregulated miRNAs in MS vs. EC; (**B**) graphical representation of the miRNA–gene network of downregulated miRNAs in MS vs. EC; and (**C**) Venn diagram of genes regulated by downregulated miRNAs and upregulated miRNAs.

Figure 4A is the graphical representation of the miRNA–gene network for genes regulated by downregulated miRNAs in Medical Samples versus External Controls, while Figure 4B represents the miRNA–gene network for genes regulated by upregulated miRNAs. We observed that the network regarding downregulated miRNAs in tuberculosis was much bigger and had a higher number of interactions per miRNA compared to the upregulated network, indicating that there are more genetic interactions being regulated in the External Control group than in both Tuberculosis patients and Hospital Controls. In total, 118 genes were regulated in both networks, as indicated by the Venn diagram in Figure 4C.

**Figure 5:**
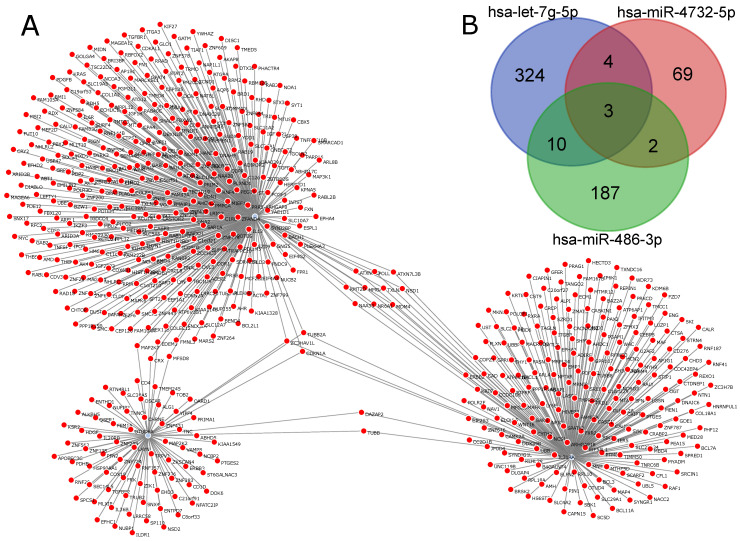
miRNA–gene network for TP vs. HC generated by ncRNA-network tool: (**A**) miRNA–gene network for the three DEmiRNAs (*hsa-miR-4732-5p*, *hsa-miR-486-3p* and *hsa-let-7g-5p*); and (**B**) Venn diagram indicating intersections between genes for each miRNA.

For Tuberculosis patients versus Hospital Controls, we used as input for the ncRNA-network tool a total of 599 different genes. Figure 5A represents the miRNA–gene network for this comparison. Among the three DEmiRNAs, an interaction overlap of three genes, *CDKN1A*, *ZC3HAV1L* and *TUBB2A*, was found (Figure 5B). Interestingly, *hsa-let-7g-5p*, which had a higher expression in Tuberculosis patients, also had the highest number of regulated genes in this comparison.

## 3. Materials and Methods

### 3.1. Sample Collection

The present study consisted of 22 samples distributed in three groups (Tuberculosis patients, 8 samples; Hospital Control, 6 samples; and External Control, 7 samples).

The Tuberculosis patients group was obtained from a set of patients with active pulmonary tuberculosis in regular treatment using standard tuberculosis treatment protocols in Brazil [36]. Eligibility criteria for Tuberculosis patients group include: (i) being over 18 years old; (ii) on sputum smear examination results for acid-fast bacilli and/or the culture of *Mycobacterium tuberculosis*; (iii) manifesting respiratory symptoms (persistent productive cough, fever and weight loss); (iv) chest radiography/computed tomography images compatible with a specific pulmonary process; and (v) confirmation by Rapid Molecular Test for tuberculosis (Xpert^®^ MTB/RIF). Tuberculosis patient samples were obtained from patients attending the Pulmonary Tuberculosis Clinic of the Hospital Universitário João Barros Barreto (Universidade Federal do Pará, UFPA). All Tuberculosis patient samples were collected directly by their attending physician.

Hospital Control samples were collected from health professionals of the same clinic, annually performing physical examinations and TST tests (advocated by the Ministry of Healthy, Brazil) and had no history of tuberculosis over 15 years of activity. Hospital Control was composed by health professionals without prior history of tuberculosis and that had constant contact (at least 4 h/day workload) with patients with active tuberculosis.

The External Control group consisted of volunteers without tuberculosis and no contact with Tuberculosis patients in treatment. External Control samples were acquired from employees of other departments from the same university, who had no contact with the university’s health services and underwent clinical evaluation.

Biological material was collected in a 5 mL tube containing RNA later and stored until RNA extraction. Samples from all groups were retrieved during 17–22 May 2017. The data were collected after the research was explained and the patients signed an informed consent form.

### 3.2. RNA Extraction and Quantification

Peripheral blood samples (5 mL) were collected using Tempus Blood RNA Tube (Thermo Fisher Scientific, Waltham, MA, USA) and stored at −20 °C until extraction. Total RNA was extracted using MagMAX RNA Isolation Kit (Thermo Fisher Scientific, USA) and quantified with NanoDrop-1000 spectrophotometer (Thermo Fisher Scientific, USA). Agilent RNA ScreenTape assay and 2200 TapeStation Instrument (Agilent Technologies, USA) were used to detect and ensure RNA integrity.

### 3.3. Library Construction and Sequencing

For small RNA-Seq, 1 μg of total RNA per sample was used for library preparation using TruSeq Small RNA Sample Prep Kits (Illumina, San Diego, CA, USA). Size-distribution was measured with the DNA ScreenTape assay on a 2200 TapeStation system (AgilentTechnologies, US). A total library pool of 4 nM was sequenced using a MiSeq Reagent Kit v3 150 cycle on a MiSeq System (Illumina, San Diego, CA, USA).

### 3.4. miRNA Quantification and Normalization

Biological data obtained from the 22 sequenced samples underwent a quality control pipeline to remove adapters used during sequencing, as well as to trim and filter the obtained sequences. This quality control process was performed using Trimmomatic software version 0.36, “ILLUMINACLIP” (with a custom adapters list), “LEADING:10” and “TRAILING:10” (both cut bases with quality lower than 10 at the beginning or end of the read, respectively), “SLIDINGWINDOW:3:22” (performs a sliding window approach with a three bases window size, cutting bases once the average quality within the window falls below 22) and “MINLEN:16” (remove reads with less than 16 bases) parameters were used. Sequences were then aligned with the human genome (HG19) using STAR software. Result files, generated as “.sam” format by STAR, were manipulated using samtools and converted to “.bam” files.

miRNA expression quantification was performed with HTSeq software, using the human genome annotation file (“.gff”) and “type” parameter set to “miRNA”. Data from alignment process were properly classified before being submitted to a new quality control, which kept miRNAs with at least 10 total read count in at least one sample and removed samples with a total read count lower than 1000. This procedure resulted in the removal of one sample from the analysis (sample belonging to TB group); this new data served as the raw data for the differential expression analysis.

Two different types of normalization were employed, differential expression analysis was performed using edge package function “calcNormFactors” (which utilizes TMM normalization method as package default). For other analysis such as histograms, boxplots and principal component analysis, raw data was normalised by counts per million (CPM), a measurement of read abundance used to compare the expression of miRNA in different samples or libraries sizes.

### 3.5. Differential Expression Analysis

Exploratory data analysis was performed with R version 3.5.0, RStudio (v1.1) and shell script. Since samples were divided into three different groups, five differential expression analyses were performed comparing: (i) Tuberculosis patients versus Controls (both Hospital and External Control groups); (ii) Tuberculosis patients versus Hospital Controls; (iii) Tuberculosis patients versus External Controls; (iv) Hospital Controls versus External Controls; and (v) Medical Samples (formed by both Tuberculosis patients and Hospital Control groups) versus External Controls. Differential expression analysis was performed with *edgeR* package that implements a statistical method for negative binomial distribution analysis. Thus, negative binomial models were used to capture the quadratic mean-variance in RNA-seq data and FDR adjustment method was applied for multiple comparison corrections. miRNAs with adjusted *p*-value < 0.05 and |FC|>1 were isolated and considered with differential expression.

### 3.6. Modeling a miRNA–Gene Networks and Analysis

Interactions between miRNAs and their target genes are extremely complex, so it is necessary to implement computational methods to allow their better understanding. Thus, network modeling is a valuable approach to measure and visualize interactions between different components of regulatory networks. We modeled a network of miRNA–gene interaction based on public data of miRTarBase [37]. miRTarBase has catalogued 300,000 interactions between miRNAs and genes, which are validated by different types of experimental studies including microarray data, western blot, report assays and next-generation sequencing.

Our miRNA network was modeled as a bipartite graph G = (V, U, E), in which G is a graph comprised by two distinct sets of regulatory elements: U is the set of miRNAs and V is the set of genes. Interactions between miRNA and genes were defined if there are two or more experimentally validation studies based on evidences catalogued in miRTarBase.

For the miRNA–gene network, we computed the number of interactions (degree of a node) as a centrality index for both regulatory elements, genes and miRNAs. After differential expression analysis, we classified DEmiRNAs on the network as upregulated or downregulated miRNAs to investigate patterns of interactions between both distinct groups.

The networks were constructed and graphically represented with NetworkX implemented in Python 3 and an in-house tool available at www.lghm.ufpa.br/ncrnas, accessed on 5 December 2020.

## 4. Conclusions

Our results identify 153 DEmiRNAs among all comparisons. The differential expression analysis between Tuberculosis patients versus Hospital Controls revealed three DEmiRNAs (*hsa-miR-4732-5p*, *hsa-miR-486-3p* and *hsa-let-7g-5p*, which was previously associated with regulation of apoptosis). These three DEmiRNAs are our first suggestion of potential biomarkers for active tuberculosis. However, validation with larger sample numbers are still required.

Our analysis indicates that only a small group of miRNAs were potentially associated with absence of active tuberculosis in healthy physicians. The presented results show healthy physicians with highly similar miRNA expression levels to tuberculosis patients. This raises concerns about the efficiency of the current safety measures employed in long-term exposure to tuberculosis environments.

Our findings provide a vast number of DEmiRNAs, which can be further studied to provide better insight into the mechanisms involved in host response to tuberculosis as well as for discovering other biomarkers for the disease. Our results can continue to be explored in other molecular research involving genetic interactions in tuberculosis, such as differential co-expression analysis or to re-evaluate safety measures regarding long-term exposure to tuberculosis currently applied in medical centers.

## 5. Study Limitations

There were some limitations to this work: (i) the small sample number could hide important DEmiRNAs; and (ii) hospital and external control groups were not tested for IGRA to denote any infection. However, this is the first work to bring data related to health professionals in daily contact with high bacillary loads of tuberculosis, which could indicate possible biomarkers of better diagnostic accuracy, besides proposing a new mechanism of labor monitoring of these professionals regarding the risk of manifesting the disease.

## Figures and Tables

**Table 1:** Gene set enrichment analysis results for genes associated with upregulated miRNAs (Medical Samples vs. External Control analysis).

Pathway	Entities Found	FDR Value
Cytokine signaling in immune system	82	6.2 × 10^−14^
Apoptosis	23	1.55 × 10^−8^
FOXO-mediated transcription of cell cycle genes	8	8.98 × 10^−6^
TP53 regulates transcription of cell death genes	11	9.55 × 10^−5^
Signaling by TGF-β Receptor complex	9	1.87 × 10^−3^
Transcriptional regulation of pluripotent stem cells	6	4.48 × 10^−3^
FcϵRI signaling	10	1.04 × 10^−1^
Interleukin-6 signaling	2	1.34 × 10^−1^
Adaptive immune system	30	1.64 × 10^−1^
Innate immune system	37	1.64 × 10^−1^
Regulation of TLR by endogenous ligand	2	1.64 × 10^−1^
Signaling by B Cell Receptor	7	1.64 × 10^−1^
Autophagy	6	1.95 × 10^−1^
Metabolism of nitric oxide: NOS3 activation and regulation	1	6.17 × 10^−1^
TNF signaling	1	6.97 × 10^−1^
FcγR dependent phagocytosis	3	8.29 × 10^−1^
Antimicrobial peptides	1	9.44 × 10^−1^
ADORA2B mediated anti-inflammatory cytokines production	1	9.76 × 10^−1^
Metabolism of lipids	19	9.99 × 10^−1^

**Table 2:** Gene set enrichment analysis results for genes associated with downregulated miRNAs (Medical Samples vs. External Controls).

Pathway	Entities Found	FDR Value
Cytokine Signaling in Immune system	278	2.11 × 10^−14^
FOXO-mediated transcription of cell cycle genes	23	6.02 × 10^−13^
Signaling by TGF-β Receptor complex	34	3.67 × 10^−10^
Transcriptional regulation of pluripotent stem cells	21	9.86 × 10^−8^
Apoptosis	44	1.39 × 10^−6^
TP53 regulates transcription of cell death genes	23	2.07 × 10^−6^
Interleukin-6 signaling	10	1.23 × 10^−4^
FcϵRI signaling	35	2.17 × 10^−2^
TNF signaling	11	2.55 × 10^−2^
Regulation of TLR by endogenous ligand	8	2.85 × 10^−2^
Autophagy	25	4.44 × 10^−2^
Signaling by B Cell Receptor	25	1.19 × 10^−1^
Innate immune system	137	2.33 × 10^−1^
FcγR dependent phagocytosis	22	2.6 × 10^−1^
Adaptive immune system	97	3.6 × 10^−1^
Metabolism of nitric oxide: NOS3 activation and regulation	3	7.34 × 10^−1^
ADORA2B mediated anti-inflammatory cytokines production	9	9.59 × 10^−1^
Antimicrobial peptides	4	9.97 × 10^−1^
Metabolism of lipids	69	1

## Data Availability

The miRNA datasets generated and analysed during the current study are available in the European Nucleotide Repository under accession PRJEB36699.
